# IRACEMA: A Database Management System for Bioactive Compounds Obtained and Characterized by Brazilian Researchers

**DOI:** 10.1021/acsomega.5c11939

**Published:** 2026-04-06

**Authors:** Thais A. Lourenço, Renan P. O. Costa, Luis Felipe M. Melo, Uilames A. Ferreira, Eduardo Henrique P. Alves, Luciano P. Oliveira-Filho, Luciana Scotti, Marcus Tullius Scotti, Gustavo Henrique G. Trossini

**Affiliations:** † Laboratório de Integração entre Técnicas Experimentais e Computacionais (LITEC), Faculdade de Ciências Farmacêuticas, Universidade de São Paulo, São Paulo, São Paulo 05508-000, Brazil; ‡ Laboratório de Quimioinformática, Instituto de Pesquisa em Fármacos e Medicamentos, 28097 Universidade Federal da Paraíba, João Pessoa, Paraíba 58050-585, Brazil; § Instituto de Estudos Avançados, Universidade de São Paulo, São Paulo, São Paulo 05508-000, Brazil

## Abstract

Bioactive compounds are natural or synthetic substances with regulatory roles in metabolic processes. Brazil, known for its rich biodiversity and strong expertise in organic chemistry, holds great potential for discovering bioactive molecules. However, the vast amount of data generated in this field is often scattered and difficult to access. The IRACEMA (Innovative Research, Analysis, and Computational Exploration of Molecules Assembled in Brazil) project addresses this challenge by establishing the first comprehensive database of synthetic bioactive compounds in Brazil. This platform integrates biological activity data, manually curated from the literature, with cheminformatics-based predictions of physicochemical and ADME properties to facilitate the exploration of structure–activity relationships (SARs) by end users. The project’s technical architecture employs modern web technologies for both frontend (React) and backend (NestJS/Node.js and Python/Flask microservices) development. The system delivers an interactive platform for molecular visualization and analysis, with PostgreSQL and Prisma ensuring robust data management. By democratizing access to data, IRACEMA (available at https://iracema.fcf.usp.br/) strengthens Brazil’s position in bioactive compound research, bridging the gap between academic discoveries and real-world applications.

## Graphical abstract

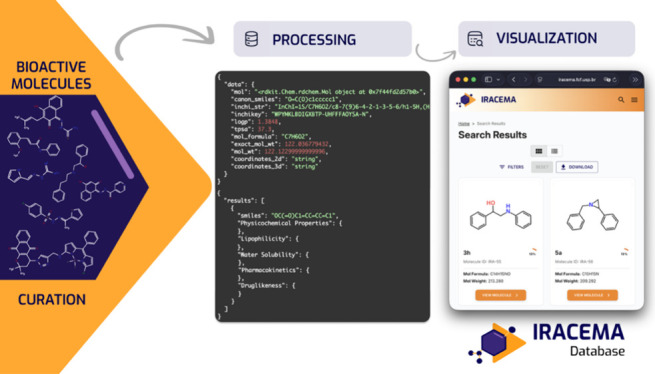

## 1 Introduction

Bioactive compounds are a vast class of chemical substances found in nature or synthesized in a laboratory that can cause biological (pharmacological or toxicological) responses in cells, tissues, or organisms.^1^ These compounds are characterized and classified based on their molecular structure and chemical properties, which influence their bioactivity and potential applications.^2–4^ The unique and diverse chemical structures make them valuable starting points for medicinal chemistry and drug development.^4,5^ Brazil, known for its rich biodiversity and strong expertise in organic chemistry, holds great potential for research and development of these molecules.^6^

The research and development of bioactive compounds, both in Brazil and worldwide, have driven a substantial increase in the amount of available chemical and biological data, transitioning between public and private sources. This growth in data, aligned with the search for new effective molecules, leads us to the concept of chemical space. This term refers to the theoretical universe of all possible molecules and their characteristics, and to improve our understanding, it is necessary to develop visualization and dimensionality reduction techniques.^7,8^

The need to handle this “Big Data” generated by various increasingly automated approaches has encouraged the emergence of cheminformatics.^9^ The field of cheminformatics applies computational methods to solve complex chemical problems and handle large volumes of chemical data, using statistical or mathematical methods and artificial intelligence to develop predictive models of molecular properties and assess molecular similarities.^10–13^ Although this approach is essential, the sheer size and complexity of the resulting data sets create significant challenges that are amplified by the four Vs (Volume, Variety, Velocity, and Veracity) and the value of the data. Furthermore, significant information is lost due to issues such as errors in data creation and manipulation.^10,12,13^ To face these challenges, the presence of organized and accessible chemical databases on the international landscape, such as ChemSpider from the Royal Society of Chemistry and SciFinder from Chemical Abstracts Service (CAS), has become indispensable for centralizing this information.^14,15^

By definition, a database is a system optimized for efficient data search, query, and management, enabling the analysis of structured data and the relationships between information. On the other hand, repositories are essentially storage spaces with the purpose of preserving research data in the long term.^10^

In the context of bioactive substances and on a global scale, we have two reference databases: PubChem and ChEMBL. ChEMBL is one of the largest and most comprehensive databases for compounds with drug-like properties worldwide. It contains detailed bioactivity information for more than 2.4 million compounds manually selected from the literature and deposited data sets.^16^ PubChem is a broader resource providing access to chemical structures, properties, and biological activities across diverse sources.^17^ However, neither database prioritizes the geographical origin of compounds or their association with specific national research groups, and only a very small fraction of their content corresponds to synthetic molecules developed in Brazil.^15–18^ This limitation hinders searches that explicitly target compounds obtained or characterized by Brazilian laboratories, which in turn can reduce the national and international visibility of this research and limit collaboration between national drug design research groups.^15^

In Brazil, the scientific community of natural products and medicinal chemistry has access to key databases such as the Brazilian Biodiversity Natural Products Database (BrNPDB) and SISTEMAT eXtended Webservices (SistematX). The BrNPDB initiative, based on the previous NuBBEDB (Nuclei of Bioassays, Biosynthesis, and Ecophysiology of Natural Products Database), consists of a web platform with chemical, biological, and pharmacological data of compounds isolated from Brazilian biodiversity.^19,20^ However, the natural products available in this database may or may not have documented biological activities. Similarly, SistematX is a platform that offers a robust collection of web-based solutions. This database was developed to provide information for studies on dereplication, chemosystematics, botanical correlation, and biodiscovery of bioactive compounds, yet with a focus exclusively on natural products.^21,22^

While Brazil has robust databases for natural products, such as BrNPDB and SistematX, these platforms focus primarily on compounds isolated from Brazilian biodiversity and may or may not include documented biological activity for all entries.^19–22^ In contrast, the landscape for synthetic compounds has been comparatively underdeveloped. The recent initiative, Brazilian Compound Library (BraCoLi), was created to bring together bioactive compounds synthesized and characterized by Brazilian research groups into a downloadable repository. Despite being an excellent initiative, the current version does not provide an online query interface with flexible search and filtering capabilities.^23^

In order to add further value to the already existing network of chemical information, the IRACEMA project was conceived as the first national database focused on biologically active compounds synthesized in Brazil, featuring a user-friendly web interface. This platform aims to increase the national and international visibility of Brazilian research and facilitate collaboration among drug discovery and medicinal chemistry groups by updating the Brazilian synthesis landscape. IRACEMA integrates manually curated bioactivity data with cheminformatics tools for molecular analysis, providing predicted physicochemical and ADME properties directly associated with each compound. By democratizing access to these data and tools, IRACEMA supports the community in exploring structure–activity relationships (SARs) and identifying promising candidates, while remaining complementary to global resources such as ChEMBL and PubChem.^13,16,18^

## 2 Materials and Methods

### 2.1 Data Curation

Data curation for the current version of IRACEMA was conducted using a structured, multistep workflow designed specifically for synthetic bioactive compounds, in close collaboration with partner synthesis laboratories. This workflow was developed with the purpose of capturing, normalizing, and relating the available chemical and biological information. At the same time, its design followed recognized best practices for research data management and the FAIR (Findable, Accessible, Interoperable, Reusable) principles, ensuring traceability, reusability, and interoperability of the curated information.^12,24,25^

In line with recent guidelines for effective data curation in translational research, the process separates the steps of source organization, article screening, structured extraction of variables, cleaning and standardization, and enrichment of metadata. In the first step (organization), source articles were collected and organized according to predefined inclusion criteria:

- The article must characterize and report the measured bioactivity (e.g., IC50, EC50, LC50, etc.) of at least one synthetic organic compound.
- The bioactivity of the substances must be characterized using reliable analytical techniques, such as enzyme assays, kinetic studies, and experimental biological evaluation.
- The work must have been conducted and published by Brazilian researchers from the designated partner institutions or laboratories.
- The study was published in a peer-reviewed scientific journal.

For each selected article, all compounds with reported biological activity were identified and marked for detailed extraction (screening). During the curation step, molecules were evaluated; those with duplicate entries, no biological activity, or incorrect molecular structures were excluded. For each remaining compound, chemical and biological data were extracted (curation), including: source (laboratory name, institution name, country, state, and city), publication (journal name, year, volume, issue, first and last pages, and DOI), identifiers (molecule name and class), and bioactivity data (activity name, value, unit, target name, organism name, activity relation, and activity description). To ensure that the compounds could be used in subsequent prediction models and for structure visualization on the platform, their Simplified Molecular Input Line Entry System (SMILES) strings were generated by drawing each molecule using Marvin JS. The final cleaning and standardization stage involved data cleansing to ensure consistency and prepare the information for management. The compiled information was entered into a spreadsheet for subsequent management by PostgreSQL. The data curation process, from organizing sources and screening articles to molecule bioactivity extraction, followed the steps shown in Scheme 1.

**Scheme 1:**
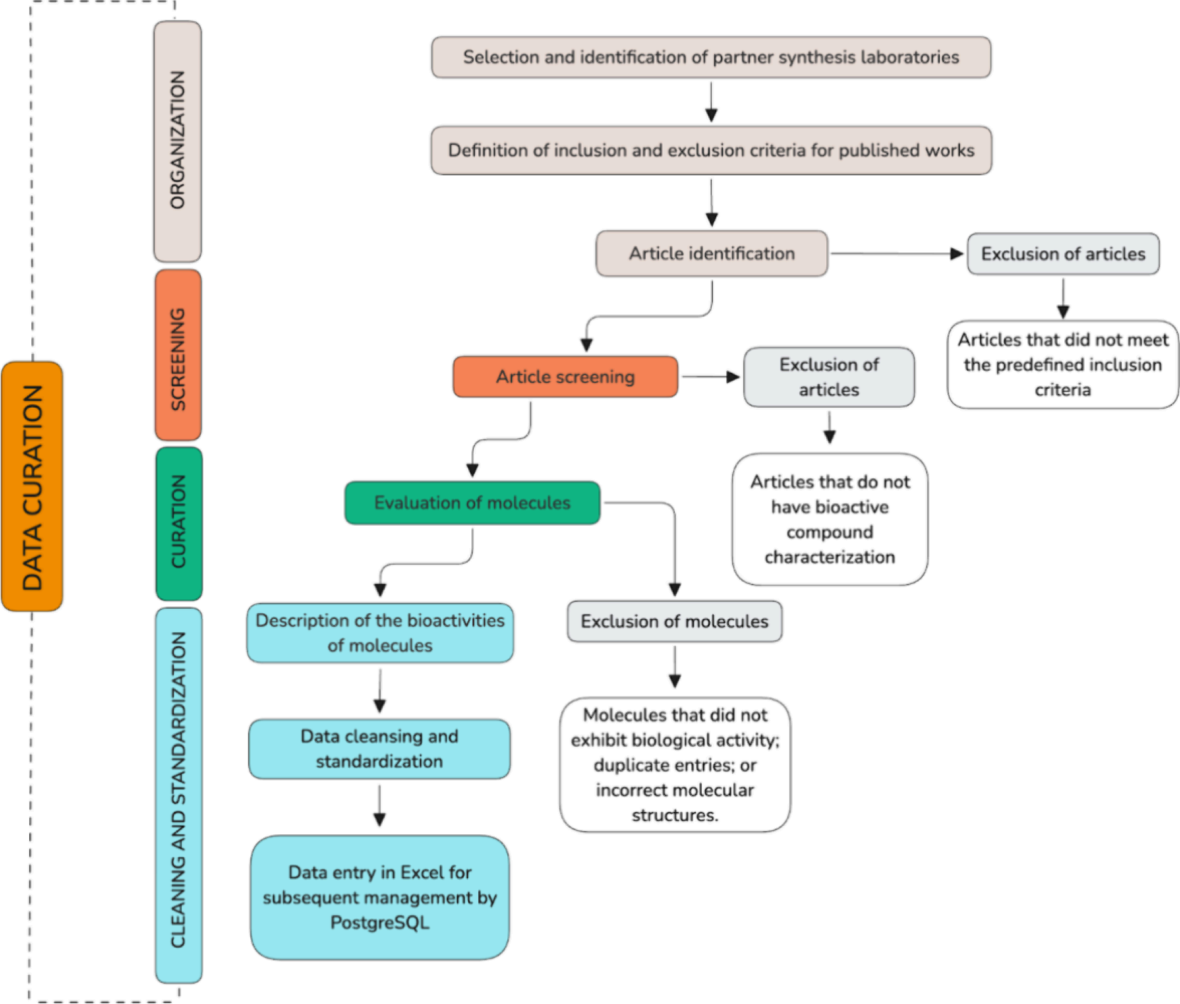
Flowchart of the Data Curation Methodology

Given that a single molecule can display multiple biological activities against distinct targets, the data model was designed so that each record corresponds to a unique biological activity (molecule–target–organism–assay). This curation process is continuous, and the same workflow depicted in Scheme 1 is being applied as new articles and laboratories are incorporated, ensuring that the database is consistently updated and its chemical space progressively expanded.^12^

### 2.2 Visual Identity and UX/UI Design

The project, named IRACEMA, was inspired by the main character from the book that bears the same name, written by Brazilian author José de Alencar. The character represents a native woman, and her name serves as an anagram for “America”, while the acronym I.R.A.C.E.M.A. (Innovative Research, Analysis and Computational Exploration of Molecules Assembled in Brazil) reflects the project’s mission. The User Experience (UX), User Interface (UI) design, and the color palette were inspired by themes of Brazilian nature and cultural elements, with a focus on prioritizing usability and accessibility for all users, particularly those with visual disabilities. Figma, combined with the Material UI framework, was used for visual implementation, ensuring consistency and agility in frontend development.^26,27^

### 2.3 System Implementation

The architecture was designed using a microservice architecture to ensure modularity, scalability, and ease of maintenance.^28^ The frontend was implemented using React (v18.3.1), with Material UI (v7.0.0).^27,29^ Interactive molecular drawings and visualizations were enabled by the integration of Ketcher (v3.2.0) and ChemDoodle (v10.0.0).^30,31^

The backend was developed using NestJS (v10.4.8) and Node.js (v22.19) in a Backend-for-Frontend (BFF) pattern, centralizing communication between the user interface, database, and specialized Python (v3.11.9) and Flask (v3.0.2) microservices.^28,32–34^ These microservices handle cheminformatics operations, leveraging RDKit (v2024.3.2) for computational processing.^28,35^ Redis (v7.0) is used for caching structural search results, improving query and pagination performance.^36^ A PostgreSQL (v16.2) database stores all curated molecular and bioactivity data, with Prisma (v5.21.1) serving as the Object–Relational Mapping (ORM) layer to streamline queries and maintain data integrity.^37–39^ The entire system is containerized using Docker, with Docker Compose (v28) orchestrating the multicontainer setup for the frontend, backend, database, and microservices.^40^ Scheme 2 summarizes the view of architectural decisions and technologies used.

**Scheme 2:**
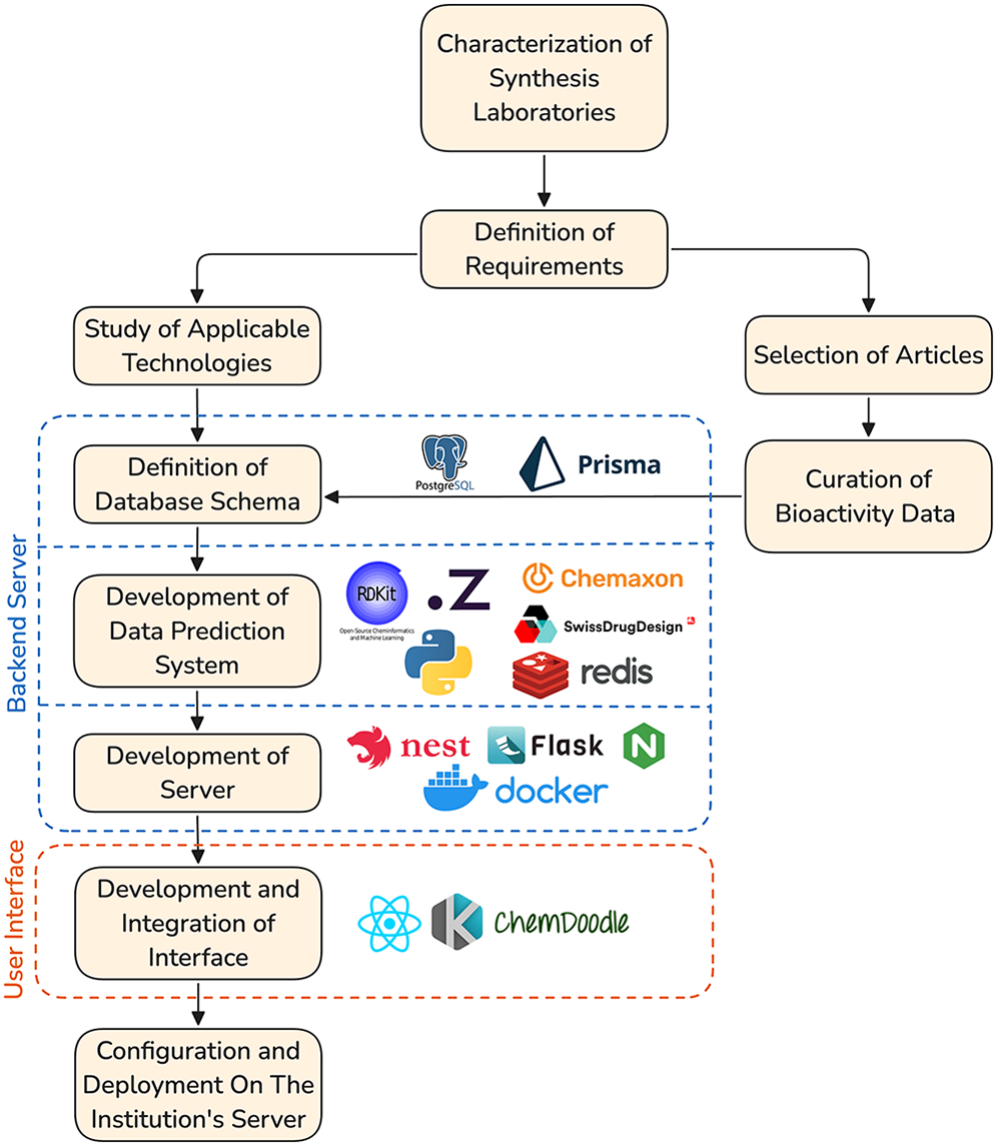
Workflow for Compound and Bioactivity Data Curation and System Architecture

The workflow for compound and bioactivity data curation is presented alongside the architectural scheme of the IRACEMA system, integrating the frontend, backend, database, and cheminformatics microservices.

This architecture ensures that cheminformatics functionalities, including molecular structure search, visualization, and access to curated bioactivity data, are delivered through a unified browser-based interface. The modular design supports continuous updates and the integration of new tools and data sets.

## 3 Results and Discussion

### 3.1 Data Curation

Data curation is an important step in building a database, as it ensures scientific integrity and promotes transparency and interoperability. The establishment of common standards, reinforced by the adoption of FAIR principles, provides a framework for organizing and sharing chemical data, thereby promoting reproducibility. Thus, this process not only improves data accessibility and sharing but also optimizes information integration and accelerates scientific discovery.^12,24,25^

The current version of the IRACEMA project focused on a curated data set from publications of selected laboratories. The synthetic substances with reported bioactivity obtained from these articles provided a manageable data set for debugging the data model, validating the curation workflow, and testing the integration of cheminformatics services while maintaining full traceability. This initial scope allowed the establishment of a robust and functional foundation for the platform.

Given that IRACEMA is designed as a dynamic database, the same curation pipeline is now being applied to additional literature and partner groups so that the number of compounds and bioactivity records is progressively expanded and the covered chemical space is broadened over time, reflecting the continuously evolving research landscape in Brazil.

### 3.2 Homepage

The development of the IRACEMA platform focused on creating a user-friendly web interface that facilitates access to its functionalities and data by visiting the link https://iracema.fcf.usp.br.

Upon clicking on the link, the user is directed to the IRACEMA landing page. A navigation menu at the top, with scroll buttons, allows transitions between different sections of the site. The “About” section provides information and guidance on using the platform, while the “Tools” section details the integrated cheminformatics tools used to draw and visualize molecular structures and predict molecular properties. Information about the research groups involved can be found in the “Team” section, and “Docs” will gather statistical data on the content of the database and its sources. The landing page also features a search bar, where users can perform specific and advanced searches for bioactive compounds, as shown in Figure 1. At the bottom of the page, a quick menu provides contact details and a link to download the complete database as two CSV files. One file contains all molecules and a summary of their available data, while the other lists all individual curated activities.

**Figure 1:**
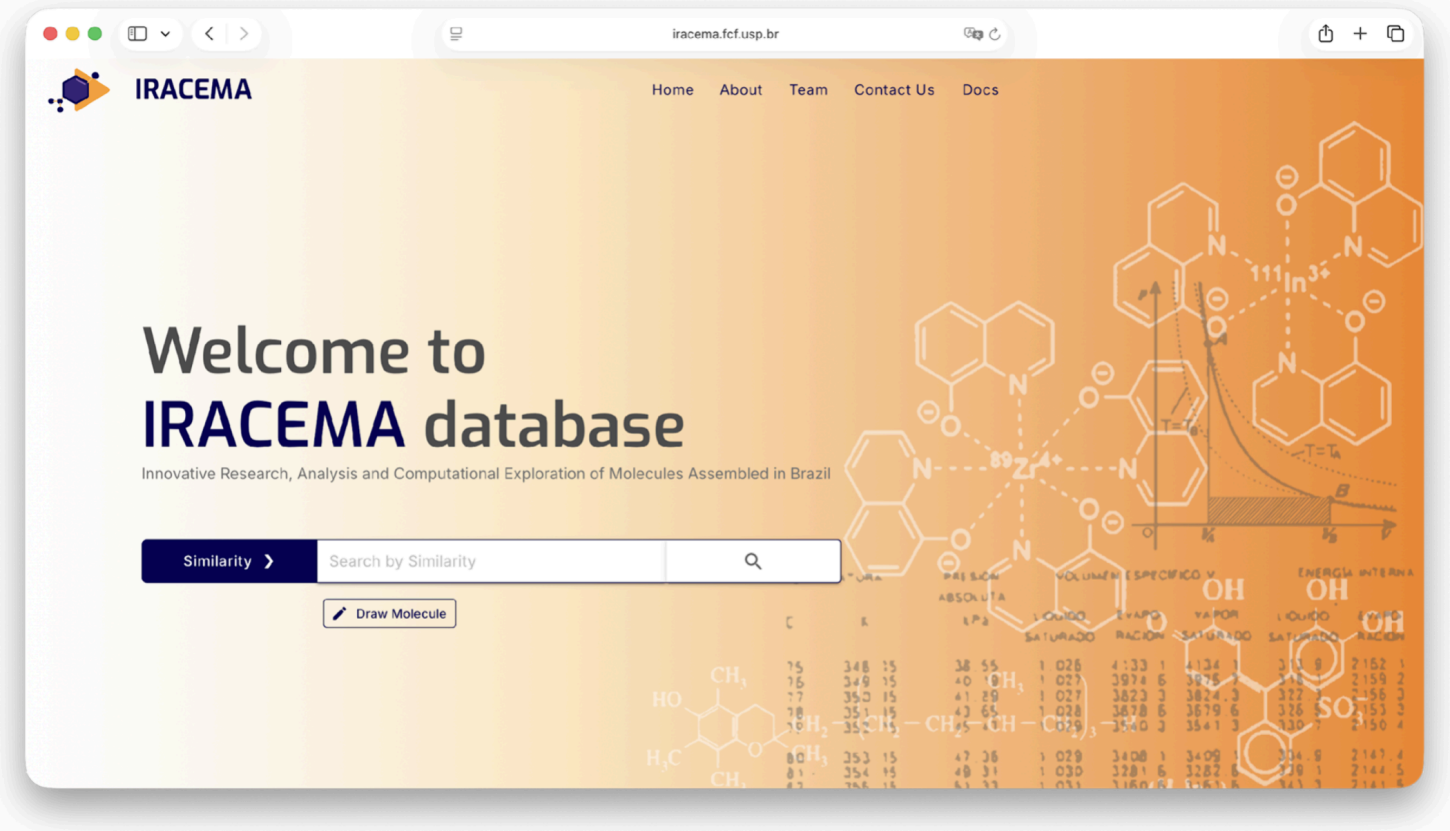
IRACEMA homepage exhibits a navigation menu at the top and a search bar in the center, where users can search for bioactive compounds using different parameters.

### 3.3 Search and Filtering Capabilities

Parameters based on the structure, targets, and source were chosen for searching and filtering. Substructure and similarity searches were performed using a hashed ECFP4 fingerprint. The similarity search was done using the Tanimoto coefficient, receiving as a parameter a similarity index between 0 and 1.0 for input using their SMILES identifier, a .mol file, or a drawn structure. The Ketcher interface, used for drawing molecules and performing structural searches, is shown in Figure 2.^28^ Furthermore, a nonstructural search uses molecule class, organism, biological target, and laboratory as filter queries.

**Figure 2:**
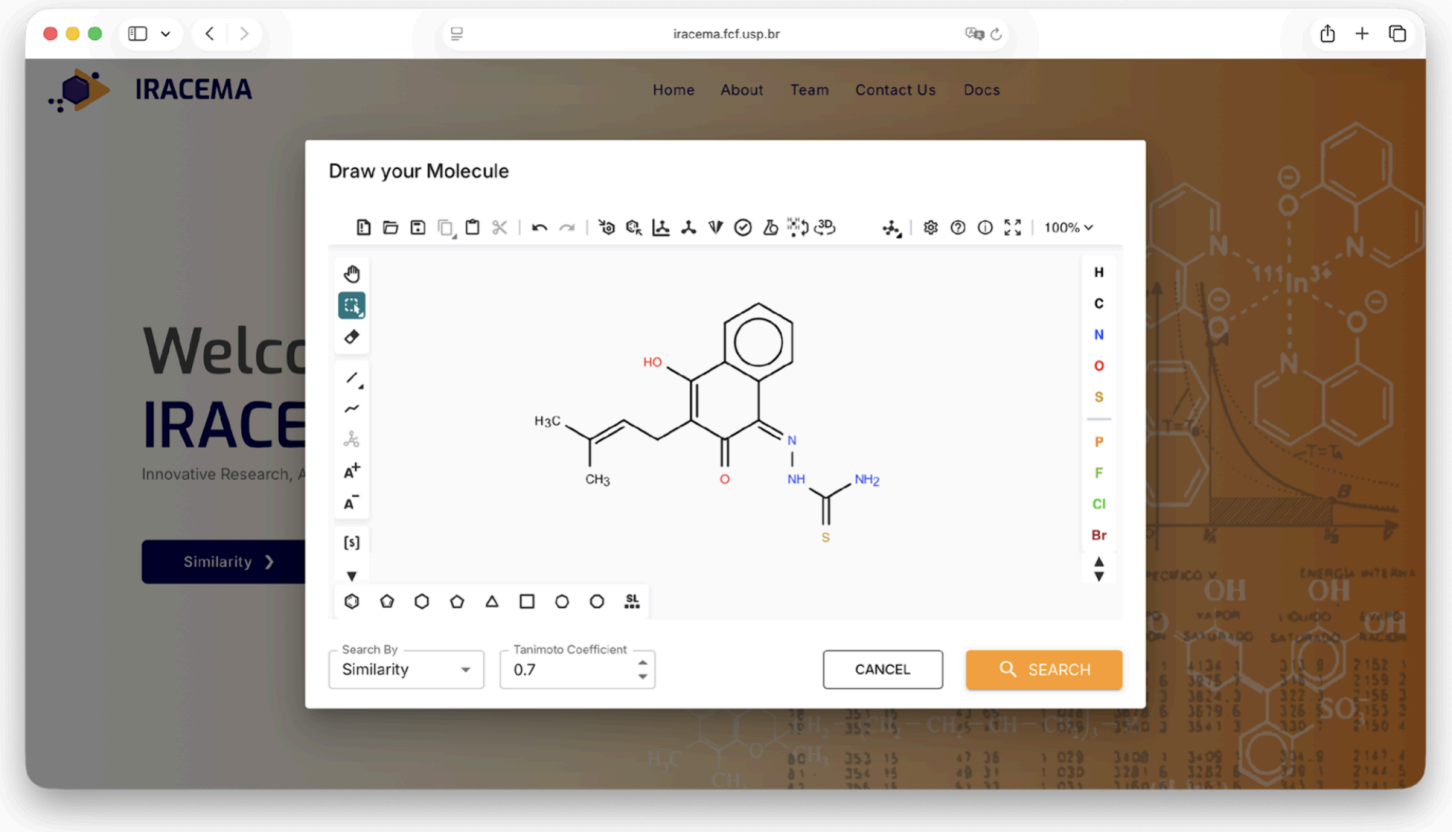
Interface of the Ketcher^28^ applet on IRACEMA displaying the structure of the following molecule: [(1*Z*)-4-hydroxy-3-(3-methylbut-2-en-1-yl)-2-oxo-1,2-dihydronaphthalen-1-ylidene]­aminothiourea. The interface allows for both substructure and molecular similarity searches (using the Tanimoto index).

Upon conducting a search, users are redirected to the results page, where the structures of interest are displayed on the basis of the search criteria, as shown in Figure 3. For a similarity search, molecules are presented in descending order of similarity. For all other search types, including structure searches and nonstructure searches, the compounds are displayed as a list of all matching entries, sorted by ID. Up to nine compounds appear on each page, and users can further refine these results by applying various filters. These include properties such as molecular weight, Wildman–Crippen logP value, and publication year, which are provided as numerical ranges that exclude any entries outside the specified physicochemical thresholds. Text-based filters, such as class, organism, target, and source information, utilize string-matching techniques that retrieve entries containing the specified query terms. Applying any of these filters initiates a new query on the initial results, showing only the compounds that meet the new specified criteria. Once the search is complete and refined, the results can still be sorted by molecular weight (ascending and descending) and alphabetically (ascending and descending).

**Figure 3:**
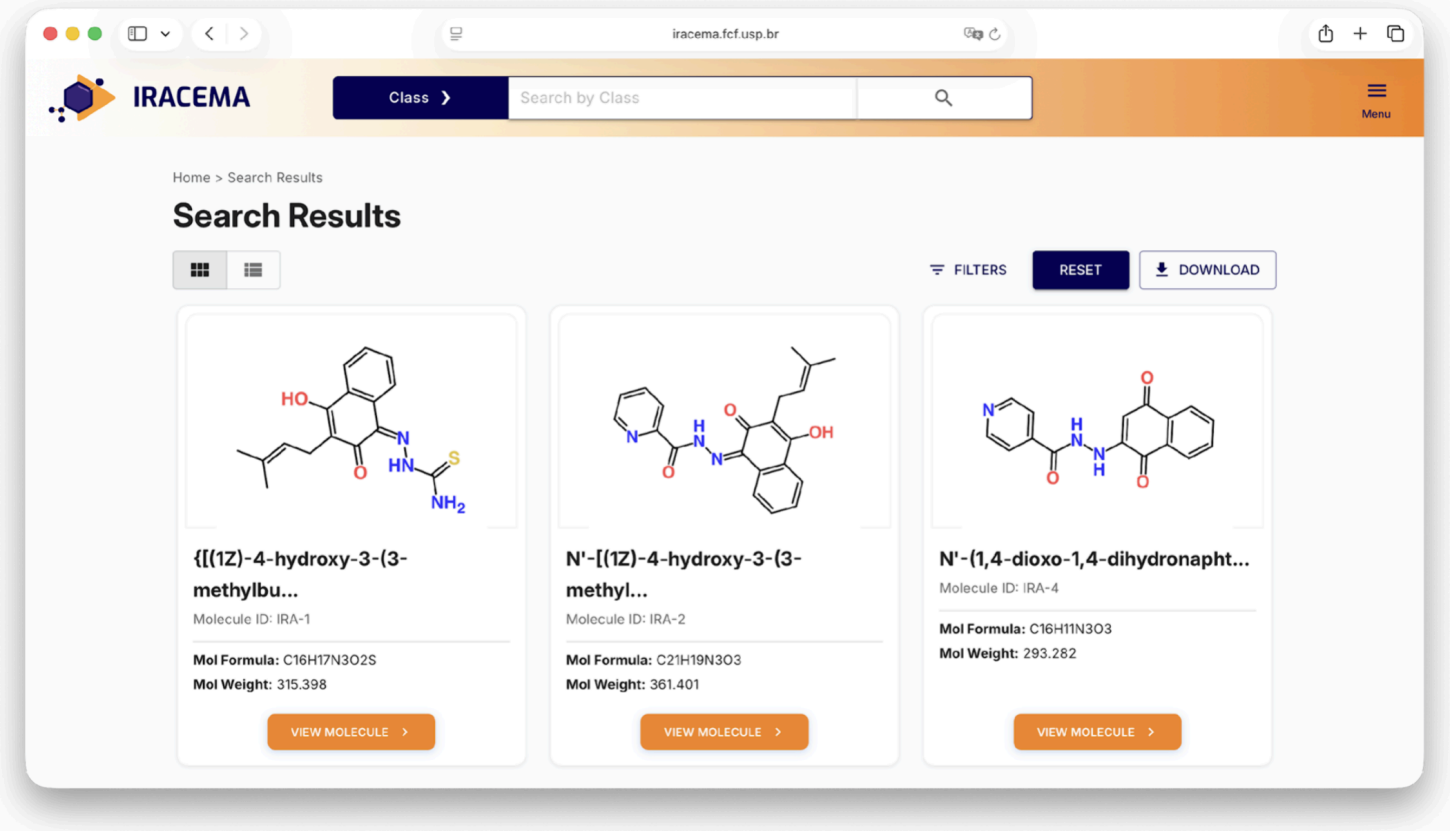
Results page displays the compounds in either gallery or list format, providing a brief description of each molecule.

Additionally, these results can be downloaded in two different formats. The SDF format can provide either 2D or 3D structural information, depending on the option selected by the user, while the CSV format offers a more detailed data set. The CSV file includes SMILES and information about the molecule’s activities, identifiers, physicochemical properties, and predicted properties, making it ideal for further analysis. These export options, together with the combination of structure-based and metadata-based filters, are consistent with the querying strategies adopted by widely used chemical databases and are intended to enable researchers to perform their own tailored analyses on top of the curated IRACEMA data.

To view detailed information about a molecule, users can click the “View Molecule” button and be redirected to that molecule’s page.

### 3.4 Molecular Analysis and Data Visualization

Upon entering a molecule’s page, users are presented with a graphical view of its structure, generated and rendered in both 2D and 3D views using ChemDoodle.^31^ This visual representation is followed by an overview of the molecule that includes its unique IRACEMA ID, key molecular descriptors, and chemical class. The initial part of the molecule page is presented in Figure 4.

**Figure 4:**
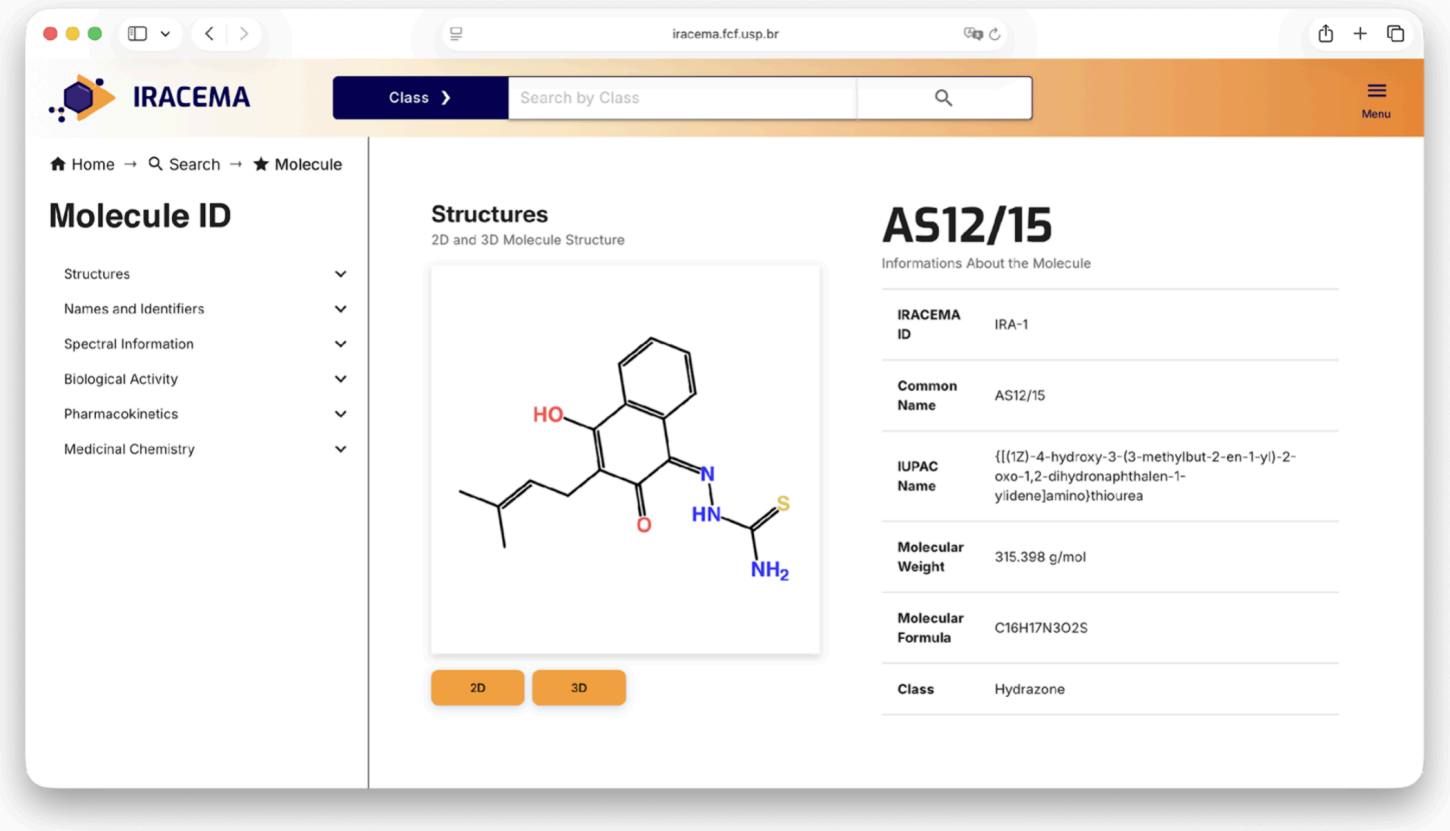
Detailed view of a selected molecule: [(1*Z*)-4-hydroxy-3-(3-methylbut-2-en-1-yl)-2-oxo 1,2-dihydronaphthalen-1-ylidene]­aminothiourea. The molecule page features 2D and 3D structural visualizations, manually curated biological activities, and predicted molecular properties.

After the structural view, the page lists the molecule’s names and identifiers. This section includes its assigned names, the International Union of Pure and Applied Chemistry (IUPAC) name, InChI and InChIKey, its CAS registry number and name, and its SMILES representation. This comprehensive list of identifiers is crucial for integrating the molecule with external databases and cross-referencing information from various sources.

A variety of physicochemical properties were calculated using established cheminformatics tools. Specifically, RDKit was employed to determine molecular descriptors such as Molecular Weight, obtained as the sum of standard average atomic weights for all atoms in the molecule, and Exact Molecular Weight (ExactMolWt), which used monoisotopic masses encoded in the molecular graph. Topological Polar Surface Area (TPSA) was computed using RDKit’s implementation of the Ertl fragment-based model, in which heteroatoms (such as N and O) are assigned to predefined fragment classes according to their local bonding environment and their tabulated contributions are summed to approximate the polar surface area. Wildman–Crippen logP (MolLogP) was derived from the Crippen module, which assigns atom-type-specific contributions to each atom and sums them to predict lipophilicity.^35,41^ These descriptors provide a compact, yet comprehensive, description of molecular size, polarity, and lipophilicity, which are directly relevant to drug discovery workflows.^9^ Additionally, the SwissADME tool was used to predict pharmacokinetic and medicinal chemistry properties, offering an ADME profile and potential drug-likeness of each compound.^42^

Last but not least, the page features information about biological activity obtained from the manual curation of published works by Brazilian researchers. This section provides a comprehensive description of the molecule’s biological activity, including the organisms, targets tested, and bioactivity assay results. In addition, each documented activity has a corresponding reference, ensuring that information for molecules tested on multiple targets, possessing various biological activities, or characterized by different laboratories is clearly stated and properly referenced.

## 4 Conclusions

This paper describes the development of the IRACEMA database, an initiative between the LITEC (USP) and the Cheminformatics Lab (UFPB) that aims to address the challenge of scattered data on bioactive compounds in Brazil. As the first database of its kind to focus on synthetic compounds in the country, it democratizes access and provides an intuitive, user-friendly platform for molecular visualization and analysis. The system uses a modern microservices architecture with a React frontend and NestJS/Node.js backend, ensuring that it is modular, scalable, and easy to maintain. By centralizing manually curated bioactivity data and integrating cheminformatics tools for the calculation and prediction of physicochemical and ADME properties, IRACEMA provides a unified environment for visualizing, filtering, and exporting information on synthetic bioactive compounds obtained and characterized by Brazilian researchers. The platform exposes high-quality, well-annotated data and derived descriptors, enabling the scientific community to build their own structure–activity or statistical models according to their research needs. This project can not only enhance the visibility of Brazilian research but also foster collaboration between local drug design research groups. The dedicated team has extensive experience in data curation and in managing similar websites, ensuring high availability, maintenance, and automated SSL certificate updates. Furthermore, the database will be continuously expanded, both in terms of the number of compounds and the breadth of the chemical space covered, ensuring that IRACEMA remains an up-to-date, reference resource for bioactive compounds synthesized in Brazil. The IRACEMA database is freely available to the scientific community at https://iracema.fcf.usp.br/.

## Abbreviations

ADME: absorption, distribution, metabolism, and excretion
BFF: backend-for-frontend
BraCoLi: The Brazilian Compound Library
BrNPDB: Brazilian Biodiversity Natural Products Database
CAS: Chemical Abstracts Service
CSV: Comma-Separated Values
DOI: Digital Object Identifier
EC_50_: half-maximal effective concentration
ECFP4: Extended Connectivity Fingerprint Version 4
FAIR: Findable, Accessible, Interoperable, Reusable
IC50: half-maximal inhibitory concentration
InChI: International Chemical Identifier
IRACEMA: Innovative Research, Analysis and Computational Exploration of Molecules Assembled in Brazil
IUPAC: International Union of Pure and Applied Chemistry
LC_50_: half-maximal lethal concentration
LITEC: Laboratório de Integração entre Técnicas Experimentais e Computacionais
NuBBEDB: Nuclei of Bioassays, Biosynthesis and Ecophysiology of Natural Products Database
ORB: Object-Relational Mapping
SAR: structure–activity relationships
SDF: structure-data file
SMILES: Simplified Molecular Input Line Entry System
SistematX: SISTEMAT eXtended Webservices
TPSA: Topological Polar Surface Area
UFPB: Federal University of Paraíba
UI: User Interface
USP: University of São Paulo
UX: User Experience
